# Assessment of quality of life after endoscopic sinus surgery for chronic rhinosinusitis

**DOI:** 10.1590/S1808-86942012000200015

**Published:** 2015-10-20

**Authors:** Thiago Freire Pinto Bezerra, Jay F. Piccirillo, Marco Aurelio Fornazieri, Renata Ribeiro de Mendonca Pilan, Fabio de Rezende Pinna, Francini Grecco de Melo Padua, Richard Louis Voegels

**Affiliations:** a MD, ENT, PhD student at Faculdade de Medicina da USP; b MD, FACS (Professor of the Department of Otolaryngology-Head and Neck Surgery, Division of Clinical Outcomes Research, Washington University School of Medicine, St. Louis, MO 63110, USA); c MD, ENT (Fellow in Rhinology at HCFMUSP); d MD, ENT, PhD student at Faculdade de Medicina da USP; e PhD (Assistant Physician at HCFMUSP); f PhD, MD, ENT Faculdade de Medicina da Universidade de São Paulo; g Professor (Director of Rhinology at HCFMUSP Associate Professor at FMUSP). Faculdade de Medicina da Universidade de São Paulo

**Keywords:** chronic disease, indicators of quality of life, quality of life, sinusitis

## Abstract

Chronic rhinosinusitis is a disease of undefined etiology that significantly impacts the quality of life of its patients. Various studies carried out in countries other than Brazil have shown endoscopic sinus surgery as an effective means of treating this condition.

**Objective:**

This study aims to analyze, with the aid of SNOT-20, the association between endoscopic sinus surgery and disease-specific quality of life of Brazilian patients treated for chronic rhinosinusitis accompanied or not by nasal polyps.

**Materials and Methods:**

This prospective study enrolled patients submitted to endoscopic sinus surgery after drug therapy failed to improve their symptoms. They were assessed based on questionnaire SNOT-20p before and 12 months after surgery. Improvement on total scores and on the five items deemed more important by each patient were assessed. The study also looked into the correlation between preoperative scores and postoperative improvement and if there were any gender-related improvement differences.

**Results:**

Forty-three patients aged 44 (19), md (IQR), 65% of whom (26/43) were males. Statistically significant improvement was seen on SNOT-20 and SNOT-20(5+) and a correlation was established between preoperative scores and postoperative improved scores (*p*<0.001). No gender-related differences were observed in quality of life.

**Conclusion:**

Endoscopic sinus surgery in patients with chronic rhinosinusitis is associated with statistically significant improvements in disease-specific quality of life.

## INTRODUCTION

Rhinosinusitis (RS) is one of the most frequent complaints recorded in medical visits in North America. Some 14% of the US population is affected by this condition at an annual cost of USD 6 billion. It is one of the top reasons for prescribing antibiotics and for reduced worker productivity^1^, ^2^, ^3^. Chronic RS (CRS) cases can be divided into those in which nasal polyps are present and those in which they are not present. Patients are differentiated based on clinical examination, histopathologic findings, interleukin profile, and prognosis^4^. CRS is also closely related to endoscopic sinus surgery, as every year over 200,000 procedures are carried out on the US alone^5^. Specific instruments to assess quality of life (QoL) connected to RS have been developed given the need to better evaluate morbidity, disease progress, and therapy impact.

Adequate validation of QoL questionnaires before they are used allows for comparisons between different populations. Quality of life questionnaire SNOT-20 (Sino-Nasal Outcome Test-20) was developed in 1998 to assess quality of life as it is specifically connected to rhinosinusitis, and has since then been used in most publications^6^, ^7^. Another similar questionnaire validated in 2009 – SNOT-22 – is still targeted by some controversy^8^. The two questions added and presented as advantageous – olfactory and nasal obstruction assessment – may be much better evaluated by other instruments such as UPSIT and NOSE^9^, ^10^. Another important fact to be considered is the vastly greater possibilities of establishing comparisons between the outcomes of long term prognostic studies started with the SNOT-20.

The importance of comparing QoL between studies was shown by the “Medical Outcomes Study Short-Form 36-Item Health Survey” (SF-36), which revealed greater morbidity levels in bodily pain measurements and social function in patients with RS than in patients with congestive heart failure, angina, chronic obstructive pulmonary disease, and back pain. This study showed that the impact on patient QoL derived from RS is much greater than what is currently credited to this disease^11^.

A thorough and strict validation process for the Portuguese version of questionnaire SNOT-20 was recently published in an international journal with the participation of the author to the original questionnaire^12^. The process ensured that an adequate validation was produced, rather than a mere translation, and the participation of the questionnaire's author in all stages of the process made sure that the questionnaire's original intentions were preserved as set in the validation guidelines^13^. Another important factor to be considered is that the use of quality of life questionnaires has to be cleared by the institution responsible for the development of the original version in English, as they hold copyrights over it.

QoL measures validly and reliably connected to RS are crucial in the reassessment of RS treatment outcomes. For example, most studies showing the benefits of antibiotics used only patients' descriptive reports of symptom improvement^14^. QoL is different from one's health status. It is the unique personal experience that reflects not only one's health status, but also other factors and circumstances pertaining to the patient's life that only he/she can describe^7^.

Functional endoscopic sinus surgery (FESS) is the treatment of choice for CRS patients not responding to drug therapy^3^. To this date, no studies carried out in Brazil had looked into the association between FESS and QoL of CRS patients using a disease-specific questionnaire thoroughly adapted and validated for the Portuguese language.

This paper aims to assess the association between functional endoscopic sinus surgery and disease-specific quality of life of CRS patients through questionnaire SNOT-20p.

## MATERIALS AND METHODS

### Study design

This prospective study was carried out between February of 2008 and October of 2010 at a tertiary care center. All patients signed an informed consent form as permitted by the Hospital Ethics Committee (no. 0522/08).

### Patient sample

Patients were consecutively recruited from February of 2008 to October of 2009 and were followed for one year.

Enrollment criteria: CRS patients (as defined by EPOS 2007) with or without nasal polyps who had not improved after three months of drug therapy and referred to FESS^3^; age above 18 years; no pregnant/lactating patients; good overall health status; no systemic or localized diseases that might compromise the patient's health. Exclusion criteria: previous endoscopic sinus surgery, secondary causes for CRS (fungus ball, invasive fungal disease, granuloma, vasculitis, isolated mucocele, nasal/sinus malignant/benign tumor, congenital anomalies – primary ciliary dyskinesia, cystic fibrosis – and oroantral fistula); congenital craniofacial anomalies; and primary/secondary immune deficiency.

### Treatment

Patients underwent functional endoscopic sinus surgery as described by Messerklinger^15^, depending on the extension of their main disease. They were given no medication preoperatively.

During postoperative care, all patients were given amoxicillin/clavulanate potassium 500mg/125mg every 8 hours for 14 days, topical steroids, budesonide spray 64 mcg on each nostril every 12 hours for 12 months. Patients also used a nasal isotonic saline solution (NaCl 0.9%) 20 ml on each nostril every 6 hours until the surgical wound was completely healed and no crust was seen in the nasal cavity under endoscopic examination.

Patients were followed up during visits with a physician on the first day after surgery, weekly for the first month after surgery and then quarterly throughout the first year. Their postoperative status was assessed based on improvements on the complaints present before surgery and bandages were put in place endoscopically with the aid of local anesthesia^16^. The patients in whom CRS still persisted one year after surgery were referred to a specific outpatient unit to be reassessed and to decide whether they should proceed with drug therapy or if a new procedure was recommended.

### Endpoints

The endpoint looked at disease-specific QoL measured as a function of the total score and the score attained in the five items deemed more relevant by each individual patient on the SNOT-20p questionnaire validated for the Portuguese language^12^ before and 12 months after surgery.

### Statistical analysis

Sample calculation was carried out considering an alpha value under 5% and a beta value under 20% for a standardized effect size of 0.5 and estimated loss of 25% with a total of 43 patients.

The data sets were analyzed using SPSS 10.0 (SPSS Inc, Chicago, IL). The Kolgmorov-Smirnov test was used to assess goodness of fit in relation to the normal distribution. The Wilcoxon signed-rank test was used to compare questionnaire scores before and after surgery. We have also assessed the size of the effect of surgery upon disease-specific QoL. The correlation between preoperative scores and postoperative improvement was assessed and calculated by the difference between the postoperative and preoperative scores using Spearman's rank correlation coefficient. We looked if there was any difference in QoL improvements in relation to gender using the Mann-Whitney U test. A p value under 5% was considered significant.

## RESULTS

Forty-three patients with chronic rhinosinusitis accompanied or not by nasal polyps submitted to FESS were included in the study; 90.7% (39/43) of them completed the study. Most patients were males [26/43 (60,5%)] with a median age of 44.0 (interquartile range (IQR) =19).

**Figure 1:**
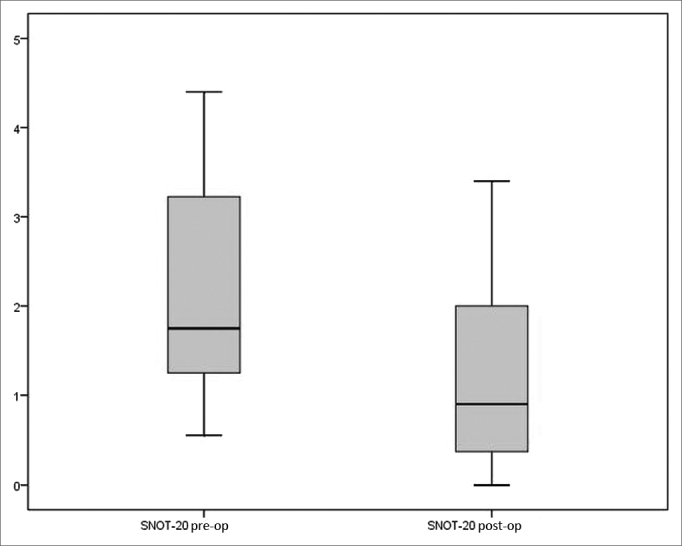
Total preoperative and postoperative scores on SNOT-20 (*p*<0.001).

A statistically significant reduction was seen between the scores attained pre and postoperatively on SNOT-20 [1.75 (IQR=2.05) *vs*. 0.90 (IQR=1.65), (*p*<0.001, Wilcoxon signed-rank test)] (Figure 1).

**Figure 2:**
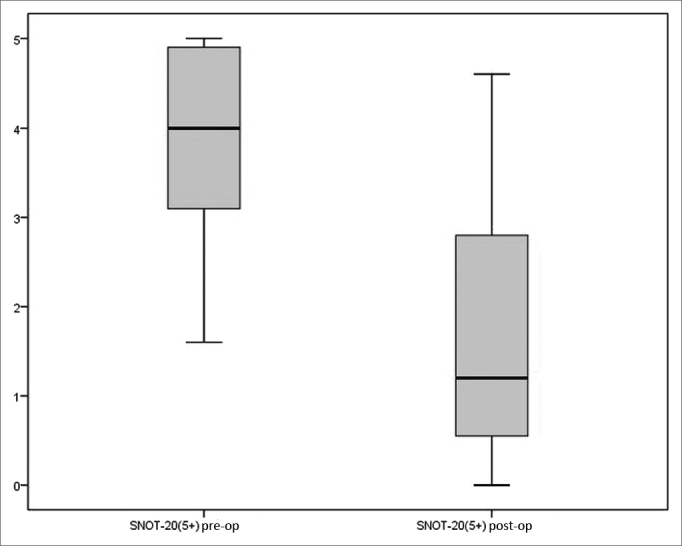
Preoperative and postoperative scores of the five items deemed more relevant by the patients on SNOT-20 (*p*<0.001).

A statistically significant reduction was also observed in the scores of the five items deemed more relevant by the patients on SNOT-20 between the pre and postoperative times [4.00 (IQR=2.00) *vs.* 1.20 (IQR=2.50), (*p*<0.001, Wilcoxon signed-rank test)] (Figure 2).

**Figure 3:**
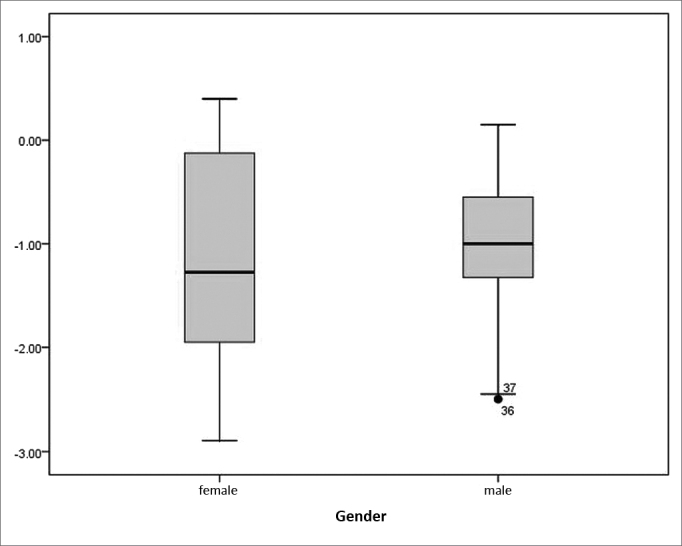
Differences in preoperative and postoperative scores on SNOT-20 between genders (*p*=0.484).

Surgery resulted in a standardized effect size of 1.13. Spearman's rank correlation coefficient showed a moderate, statistically significant correlation between the preoperative total scores on the SNOT-20 and postoperative improved scores (r=-0.547, *p*<0.001). According to the Mann-Whitney U test, no statistically significant differences were found on SNOT-20 scores of male and female patients (*p*=0.484) (Figure 3).

## DISCUSSION

Functional endoscopic sinus surgery had a positive association with CRS patients' QoL as seen in the statistically significant difference observed in SNOT-20 scores before and after surgery [1.75 (IQR=2.05) *vs*. 0.90 (IQR=1.65), (*p*<0.001)]. Our results have also shown a moderate, statistically significant correlation between higher preoperative scores and greater reduction on postoperative scores, indicative of greater impact on the QoL of the patients with the worse QoL before surgery (r=-0.547, *p*<0.001).

This study presents a few limitations. One of them is the absence of a control group to preclude bias on patient improvement being influenced by the disease's natural history in some cases or by a placebo effect in others. Nonetheless, all the patients who underwent surgery had already been submitted to longstanding drug therapy to no avail. Another is the fact that the study was carried out at a tertiary care unit. Our sample is usually made up of more complex cases. One of the ways of reducing bias was accepting only patients who had not been previously operated on.

Many are the positive things to be stressed in our study, such as its prospective design, the use of a properly adapted and validated assessment instrument, the assessment of results done from the standpoint of the patient, and a low rate of lost patients during follow-up. An important note: surgeons were blinded for preoperative SNOT-20 scores.

Many studies have shown the impact of FESS on QoL using disease-specific questionnaires, but to this date there had been no studies of this sort done in Brazil. A systematic review carried out in 2005 found a number of papers from other countries that showed improved quality of life after FESS^17^. Two large prospective studies were done after this review by Ling et al.^18^ and Bhattacharyya et al.^19^ and also showed positive impacts on QoL after surgery. A large multicenter prospective study done in the United States published recently showed that 72% to 76% of the patients had significant clinical improvements on disease-specific QoL after surgery^20^. The results from our sample of patients matched the results found by the author in the development of the original questionnaire and those of a similar study carried out in Germany^7^, ^21^. Only two clinical trials were found in the literature comparing surgery to drug therapy. Neither showed statistically significant differences. However, none of them followed the patients after the cessation of drug therapy^22^, ^23^.

Many studies done in the past to assess the effectiveness of FESS in the treatment of chronic rhinosinusitis patients used non-validated QoL questionnaires and looked into the presence or absence of certain symptoms, alterations viewed endoscopically or on CT scans^24^, ^25^, ^26^. The results of these studies could not be used to compare populations and were hard to interpret. Physical examination findings may often be biased by subjectivity or simply not be correlated to the actual status of diseased patients.

Changes on endoscopic examination of CRS patients can be correlated to QoL, although postoperative nasal/sinus improvement may explain for only a small portion of the QoL improvement seen after FESS^27^. Improvements on CRS patient symptoms or QoL after FESS are very weakly correlated to CT findings^28^.

The validated disease-specific QoL questionnaire for CRS patients – SNOT-20 – has been the most widely used of its kind in the world and has now been validated in the Portuguese language^12^. An available valid questionnaire will allow the results of the studies done in Brazil to be compared to the results of studies carried out all over the world to assess the impact on disease-specific QoL. This questionnaire is also correlated to patient global QoL (SF-36) and the visual analogue scale^28^.

There are other validated instruments to assess the impact of nasal/sinus complaints upon QoL. The RSOM-31 (Rhinosinusitis outcome measure) contains 31 questions divided into seven subsets; however, the scale onto which the answers have been placed make it somewhat hard to answer^29^. The RSDI (Rhinosinusitis Disability Index) relates nasal/sinus symptoms to specific limitations placed upon daily life activities through 30 questions in a similar fashion to the RSOM^30^. The RQLQ (Rhinoconjunctivitis quality of life questionnaire) is directed to allergy symptoms of a nasal/sinus nature, but it has not been validated for rhinosinusitis^31^.

The SNOT-20 is a simplification of the RSOM-31 in which 11 items have been removed due to redundancy or simply because they did not add significant value to the instrument. The scoring system for answers and calculation formula to obtain the result of the questionnaire were simplified. The five patient-selected items make up a second score^7^.

The recently validated^8^ SNOT-22 is made up by the simple summation of olfactory and nasal obstruction scores on the SNOT-20. It is an important tool, but we believe that in most prospective studies it would be more advantageous to assess olfaction using specific tests such as the UPSIT^10^ and to evaluate nasal obstruction using the NOSE^9^ questionnaire.

The UPSIT allows for a broader assessment of hyposmia rather than through only one question. Soter et al.^32^ have shown that the UPSIT is much superior than the simple perception patients have over their sense of smell. The completion of the validation process of the UPSIT test (“University of Pennsylvania Smell Identification Test”) for the Portuguese language, by Fornazieri et al.^10^, will allow a more objective and specific assessment of our patients' olfaction. The questionnaire that was in use in Brazil contained a few glitches, and the transcultural adaptation process will aim at producing a version that is closer to our reality.

We have been using a Portuguese-validated, accepted-for-publication version of the NOSE (Nasal Obstruction Symptom Evaluation)^9^ to assess QoL specifically connected to nasal obstruction, as it assesses complaints of this nature in a multifaceted manner. It is a free instrument. The NOSE allows the analysis of other facets pertaining to nasal obstruction, as patients are often not bothered or fail to complain specifically about it, though they mention nasal congestion or difficulty breathing through the nose. We have opted for the use of this easy-to-use instrument in our studies because it looks into such complaints more thoroughly in only two minutes. It can be used to assess any nasal disease associated with this complaint, and not only chronic rhinosinusitis.

We are not suggesting that CRS patients should not be asked these questions. We are only indicating that these two items can be analyzed in more detail through UPSIT and NOSE, instead of the simple addition of two questions. These instruments are used mainly for research, not in our daily practice; from the standpoint of research method, it would be better to use NOSE and UPSIT than merely adding these two questions.

## CONCLUSION

Functional endoscopic sinus surgery done in patients with chronic rhinosinusitis in Brazil has been associated with statistically significant improvements on disease-specific QoL for patients followed for one year. Future clinical trials carried out in Brazil will add to the scientific evidence gathered on the effectiveness of surgery offered to our population, as seen in studies performed in other countries.
